# Angiogenesis in Tissue Engineering: As Nature Intended?

**DOI:** 10.3389/fbioe.2020.00188

**Published:** 2020-03-20

**Authors:** Valeria Mastrullo, William Cathery, Eirini Velliou, Paolo Madeddu, Paola Campagnolo

**Affiliations:** ^1^Section of Cardiovascular Sciences, Department of Biochemical Sciences, University of Surrey, Guildford, United Kingdom; ^2^Experimental Cardiovascular Medicine, Bristol Heart Institute, Bristol Royal Infirmary, University of Bristol, Bristol, United Kingdom; ^3^Bioprocess and Biochemical Engineering Group (BioProChem), Department of Chemical and Process Engineering, University of Surrey, Guildford, United Kingdom

**Keywords:** scaffolds, spatio-temporal gradient, vessel maturation, angiogenesis, tissue engineering

## Abstract

Despite the steady increase in the number of studies focusing on the development of tissue engineered constructs, solutions delivered to the clinic are still limited. Specifically, the lack of mature and functional vasculature greatly limits the size and complexity of vascular scaffold models. If tissue engineering aims to replace large portions of tissue with the intention of repairing significant defects, a more thorough understanding of the mechanisms and players regulating the angiogenic process is required in the field. This review will present the current material and technological advancements addressing the imperfect formation of mature blood vessels within tissue engineered structures.

## Introduction

The early promises of tissue engineering (TE) have been slow to materialize in recent years. Despite numerous studies demonstrating the feasibility of tissue replacement with tissue engineered constructs, clinical applications are scarce (Williams, 2019). It is interesting to note how the most advanced solutions delivered to physicians are not conductive to vascularization (Fernandez de Grado et al., 2018; Williams, 2019).

Indeed, it has quickly become evident that the diffusion of oxygen and supply of nutrients is a major limit to the size and complexity of tissue engineered constructs, and that integrating a network of blood vessels represents both a necessary and challenging step.

In order to reproduce the natural vascular structure in laboratory conditions, it is of paramount importance to identify the molecular and cellular players, and their complex interactions, which determine the success of the angiogenic process.

Angiogenesis, the formation of new blood vessels from existing ones, is relatively rare in adults and almost entirely limited to areas of post-injury regeneration and tumor growth. These two types of angiogenesis are driven by similar signals, however, deliver very different outcomes. Reparative angiogenesis recreates functional and interconnected vessels, whilst tumor angiogenesis produces a high number of immature and disorganized vessels (Ziyad and Iruela-Arispe, 2011; Viallard and Larrivée, 2017).

Understanding the mechanisms regulating healthy and pathological angiogenesis should therefore provide valuable clues to generate tissue engineered constructs embedded with robust and mature vasculature.

During angiogenesis, quiescent endothelial cells (ECs) from an existing vessel are stimulated and activated by the increase in concentration of pro-angiogenic factors produced by inflammatory or tumor cells in response to injury and/or hypoxia. Activated cells proliferate and differentiate into tip cells, leading to the elongation of new vessels toward the stimulus through active migration. Stalk daughter cells ensure a continuum with the original vessel through regulated proliferation (Gerhardt et al., 2003; Carmeliet and Jain, 2011; Cathery et al., 2018). Once the capillary is formed, ECs secrete attractant molecules with the aim of recruiting perivascular cells. Perivascular cells (pericytes in the capillaries and smooth muscle cells in larger vessels) migrate along the newly formed vessels to ensheath the endothelium, providing stability, promoting cell differentiation, and regulating vessel permeability (Cathery et al., 2018).

Some key features of this mechanism are: (i) temporal regulation, (ii) spatial organization of the stimuli, (iii) cellular crosstalk, (iv) active remodeling and interaction with the extracellular matrix (ECM). When any of these features are dysregulated, the development of new vasculature is abnormal, such as in the case of tumor angiogenesis (Ziyad and Iruela-Arispe, 2011). Cancer associated vessels present excessive tortuosity, larger lumens, hyperpermeability and uncontrolled sprouting (Ziyad and Iruela-Arispe, 2011). These aberrant features are often the result of the disruption of the tightly regulated sequence of stimuli necessary to deliver healthy angiogenesis, such as the excessive or sustained release of proangiogenic factors. Similarly, angiogenesis within tissue engineered scaffolds is traditionally stimulated by the addition of single growth factors (GF) to the whole construct, leading to poorly organized and immature blood vessels, reminiscent of tumoral angiogenesis.

Therefore, this review summarizes state of the art material and TE solutions devised to overcome these current limitations, and attempts to provide new avenues to ensure the formation of mature and hierarchically organized vasculature within tissue engineered constructs.

## Temporal Control of Angiogenesis

Angiogenesis is a dynamic process where a series of events progress following a precisely timed schedule (Bentley and Chakravartula, 2017). At the single cell level, mechanical and chemical sensing drive the internal signaling response and consequent actions (proliferation, migration, and differentiation). Therefore, temporal delivery of bioactive molecules in a tissue engineered scaffold is key to the development of structured blood vessels.

Most commonly, GF and other molecules are incorporated into TE scaffolds by non-covalent adsorption in the scaffold material (King and Krebsbach, 2012). In this way, growth factor release is based on the affinity of the molecules with the scaffold material, or through controlling the kinetics of molecule diffusion (Whitaker et al., 2001). While not a time-controlled system, this method allows simple control of both abundance and diffusion of a single compound (or several compounds with different chemical affinities) released from the scaffold (Nguyen et al., 2013). This method is often used when scaffolds are employed as delivery systems for the controlled release of molecules to organs or tissues.

When the focus is instead on the delivery of molecules to cells seeded directly on the scaffold, the simplest approach is the progressive addition of molecules over time to cells in culture prior to seeding, as discussed in section “Differentiation *in vitro* (Progressive Addition of Growth Factors)”. More advanced systems have been developed using cleavable linkers and modified scaffolds to induce timed released of GF, facilitating potential use in vivo. Crucially, despite the novelty of the various delivery systems developed, the variety of molecules used to stimulate the angiogenic process remain limited (Table 1).

**TABLE 1:** List of common bioactive molecules used in tissue engineering vascularization and mentioned in this review.

Bioactive molecule	References
VEGF	Lee et al., 2001; Gerhardt et al., 2003; Chiu and Radisic, 2010; Chow et al., 2010; Yuen et al., 2010; Shah et al., 2011; Zheng et al., 2012; Brudno et al., 2013; Nguyen et al., 2013; Alsop et al., 2014; Lai et al., 2014; Rich et al., 2014; Stamati et al., 2014; Wu et al., 2016; LaValley et al., 2017; Turner et al., 2017; Kuttappan et al., 2018; Stejskalová et al., 2019; Wang et al., 2019
FGF	Chow et al., 2010; Tengood et al., 2011; Lai et al., 2014; Stamati et al., 2014; Kuttappan et al., 2018; Dong et al., 2019
IGF	Holland et al., 2005
EGF	Lai et al., 2014
PDGF	Tengood et al., 2011; Brudno et al., 2013; Lai et al., 2014; Stejskalová et al., 2019; Wang et al., 2019
Glycosaminoglycans	Chow et al., 2010; Chow et al., 2014; Wu et al., 2016

In this section, we will review the recent advances in temporally controlled release of GF in scaffolds, with a focus on the signal triggering the release of bioactive molecules.

### Differentiation *in vitro* (Progressive Addition of Growth Factors)

The most effective way to guide cell differentiation is to load bioactive molecules onto the biomaterial initially. This has been achieved by sequential or simultaneous delivery of multiple GF to pre-seeded scaffolds, leading to improved neovascularization before implantation (Tengood et al., 2011). Brudno et al. (2013) stated that the combination of VEGF and Ang2, followed by sequential addition of PDGF and/or Ang1, promoted vascular maturation *in vitro*.

However, the sequential delivery of biomolecules has several disadvantages: (i) short half-life and rapid degradation *in vivo*; (ii) uncontrollable pharmacokinetics; (iii) risk of delivering supra-physiological doses.

### Degradation Dependent Release

During sprouting, ECs produce and secrete several proteolytic enzymes which escape the ECM and migrate (Rundhaug, 2005). These enzymes are naturally able to degrade ECM-derived scaffolds used for TE, and therefore natural polymer-based scaffolds are often preferred because of their biocompatibility and biodegradation following implantation (Asghari et al., 2017). The release of molecules from the scaffold can be controlled by modifying the properties and composition of the material, as well as changing the method of retaining the drug (King and Krebsbach, 2012). Boontheekul et al. (2005) modified alginate gels, which are naturally susceptible to hydrolysis, with partial oxidation and controlled molecular weight to obtain a tunable degradation rate. Holland et al. (2005) created a system for double delivery of insulin like growth factor-1 (IGF-1) and transforming growth factor beta-1 (TGFβ-1) using oligo(poly(ethylene glycol) fumarate) (OPF) hydrogel phase and gelatin microcarriers. By controlling the degradation of the hydrogel and the microcarrier, it was possible to optimize the kinetics of the GF release (Holland et al., 2005). Similarly, Shah et al. (2011) used polyelectrolyte layers to encapsulate recombinant human bone morphogenetic protein-2 (rhBMP-2) and recombinant human vascular endothelial growth factor (rhVEGF), and showed controlled release of the encapsulated factors over time both *in vitro* and *in vivo*. Furthermore, Kuttappan et al. (2018) recently demonstrated the benefit of differential release by functionalizing a nanocomposite fibrous scaffold with combinations of VEGF, fibroblast growth factor (FGF) and BMP2. The release of these factors at different times led to increased tissue vascularization and bone regeneration (Kuttappan et al., 2018). Degradation-dependent release is based on the non-covalent adsorption of the molecules on the scaffold, and as such is susceptible to a high release of factors immediately after contact with physiological fluids, a phenomenon termed burst release (Huang and Brazel, 2001). Burst release leads to the quick loss of high concentrations of adsorbed molecules, delivering an unwanted concentration spike to the cells. Over the last decade, researchers have developed solutions to limit this phenomenon. For instance, increasing the cross-linking density of gelatin can improve molecule retention and limit its diffusion (Iwanaga et al., 2003; Patel et al., 2008). Turner et al. (2017) crosslinked gelatin microspheres to drive a more regulated release of VEGF or BMP2, dependent on the progressive proteolytic degradation of the scaffold. The non-specific degradation of the scaffold only partially controls the delivery time of GF, making it necessary to develop more advanced systems, which will be discussed later.

### Trigger Specific Release of Bioactive Molecules

Sequential delivery of GF from scaffold can be obtained by incorporation of different molecules in sequential layers of polymers, a technique termed layer-by-layer (LBL) (Rouwkema and Khademhosseini, 2016). By incorporating different biomolecules in different layers and taking advantage of matrix-degrading enzymes produced by cells, it is possible to deliver GF sequentially. One interesting example is a LBL polycaprolactone (PCL) scaffold built with layers enriched with heparin and VEGF, intended for vascular grafts applications. The authors reported an initial burst release of VEGF, triggered by the ECM degrading enzyme metallopeptidase-2 (MMP-2), followed by a controlled release of heparin with an anti-thrombogenic effect on long term vascularization of the engineered graft (Wang et al., 2019).

Advanced methods rely on the use of an enzyme sensitive linker binding the pro-angiogenic molecule covalently to the scaffold. Targeting the linker sequence to a specific enzyme (e.g., metalloproteinases, serine or cysteine proteinases) determines timely release as these enzymes are produced by cells at specific times during differentiation or angiogenesis (Fonseca et al., 2014). Alternatively, light-sensitive linkers can be used to covalently bind the molecule of interest to the scaffold, with UV or near infrared (NIR) light used to release “caged” biomolecules (Linsley and Wu, 2017; Ruskowitz and DeForest, 2018). However, the next challenge for regenerative medicine is to develop a wavelength specific photocleavable release of factors for optimal on-demand delivery of GF. Azagarsamy and Anseth (2013) tested this method by covalently binding BMP-2 and BMP-7 to a scaffold using nitrobenzyl- and coumarin-based azides linkers, respectively. The hydrogels were then seeded with human mesenchymal stem cells (hMSC), and different wavelengths of light, 365 and 405 nm, were applied to induce the sequential release of molecules, resulting in improved osteogenic differentiation *in vitro*.

A further strategy to control the release of bioactive molecules is encapsulation. Encapsulation also provides a layer of protection for GF, increasing their short half-life. Minardi et al. (2014) used this technique to design a scaffold patterned with composite microspheres, enabling spatiotemporal release of proteins. Furthermore, Lai et al. (2014) used encapsulation via nanofibers and gelatin nanoparticles to create a scaffold capable of a stagewise release pattern of VEGF, PDGF, FGF, and EGF (epithelial growth factor). The gradual release of these factors was sustained for over a month, and resulted in increased endothelial cell proliferation and development of vascular-like structures.

These novel methods offer the advantage of a trigger to control GF delivery, imitating the temporal pattern observed in natural angiogenesis, albeit with a still limited complexity.

### Mechanical Release

An alternative trigger to release GF from the scaffold is mechanic stimulation, by which cells applying traction forces can deform the scaffold at the microscopic level, liberating the entrapped molecules. A simple application of this consists of incorporating a drug into an alginate gel and allowing the cells to apply pressure on the matrix, stimulating molecule release (Lee et al., 2001). Stejskalová et al. (2019) developed a method that uses cell-specific traction forces to trigger GF release from a biomaterial construct. The technology relies on the use of Traction Force-Activated Payloads (TrAP) composed by aptamers (short, single-stranded oligonucleotides that fold into 3D structures) flanked by a cell-adhesive peptide. The aptamers trap the GF in their 3D structure, whilst the peptide is able to bind the cell surface; the whole group is anchored to the scaffold by a linker. When the cells bind to the cell-adhesive peptide and pull the scaffold structure, the cellular traction force acts as a biophysical trigger to unfold the aptamer and release/activate the GF. Importantly, delivery can be made cell-specific by selecting a peptide which binds to a cell-specific receptor. This method has been tested with VEGF on human microvascular endothelial cells (HMEC-1) and PDGF-BB on primary human dermal fibroblasts (HDFs), resulting in a remarkable increase in the proliferation rate for both cell types. Interestingly, TrAP allows the release of the payload in a temporal manner which is dependent on the expression of the cell surface receptor targeted by the adhesive peptide (Stejskalová et al., 2019).

Acoustic waves can be used to release molecules from acoustically responsive scaffolds (ARSs) (Dong et al., 2019). Dong et al. developed a bFGF functionalized ARS that released GFs upon ultrasound-mediated triggering of acoustic droplet vaporization. The authors did not observe an effect on the angiogenic sprouting following the temporal release, but the technique itself has great potential for future *in vitro* and *in vivo* applications.

## Spatial Control of Angiogenesis

In this review, we have so far discussed the temporal regulation of growth factor release during angiogenesis. However, angiogenesis *in vivo* is also tightly regulated via spatial cues that direct vessel sprouting and maturation. Stimuli such as ischemia or inflammation, provoke a localized release of GF, cytokines and chemokines, which effectively creates a gradient within the extracellular space (Carmeliet, 2000). The establishment of this molecular gradient leads to the formation of a spatially controlled leading edge of cells, which induces localized angiogenesis and increased perfusion. Current TE practices have attempted to mimic this process using simplified systems with varying degrees of success. The primary methods to obtain spatial control rely on either the direct patterning of the cells through bioprinting, or on the organized distribution of the molecules providing the pro-angiogenic stimulus.

Here, we highlight the most promising techniques and recent advancements in the field. We have limited the discussion to spatial organization of scaffolds and not the types of biomaterials used, which presents another significant consideration outside the scope of this review.

### Spatial Control of the Scaffold Architecture and Cell Organization

#### 3D Bioprinting

Three-dimensional (3D) printing has been applied extensively in the field of regenerative medicine to promote angiogenesis in engineered tissues. Through direct or indirect printing methods, cells, biomaterials, and GF can be combined to produce complex shaped constructs with defined micron-sized channels and pore sizes that are capable of guiding angiogenesis.

Direct bioprinting involves active printing of bio-ink droplets, containing cellular and extracellular components, into defined shapes. This approach requires a stringent cross-linking process, or rapid gelation of hydrogels, to produce a stable structure. On the other hand, indirect bioprinting is based on printing a sacrificial frame or channels that are then encapsulated by the cell-loaded biomaterial. These frames or channels can later be removed using thermal modifications or a suitable solvent to leave a capillary-like network, which after seeding with ECs, is used to guide angiogenesis (Sarker et al., 2018).

Inkjet bioprinting is a method of direct 3D printing which involves the LBL dispersion of bio-ink droplets onto a substrate using a thermal or piezoelectric actuator. It utilizes cross-linking agents combined with hydrogels that possess rapid gelation properties to print highly organized networks. For example, using computer aided design, alginate-based bio-inks can be printed into a calcium chloride solution where they rapidly gelate. This technique has been used to create 200 μm diameter vessels (Nishiyama et al., 2009). Furthermore, Cui and Boland (2009) demonstrated the relative ease of modifying a standard thermal inkjet printer to simultaneously print an endothelial cell and fibrin-based microvasculature. The ECs aligned and proliferated within the printed channels to form a confluent lumen-like structure (Cui and Boland, 2009). Overall, this method is inexpensive due to the ability to adapt regular printers and allows for deposition of multiple cell types. Critically, however, the stress exerted on cells during extrusion can cause apoptosis and uneven dispersion, limiting its applicability.

Extrusion or pressure assisted bioprinting is one of the most common methods used to promote angiogenesis and vasculature formation (Miri et al., 2019). This technique is similar to inkjet, however, the bio-ink is deposited using a pressure-based system rather than a thermal or piezoelectric actuator. It has been utilized by various research groups to directly fabricate vascular-like networks (Zhang et al., 2013; Jia et al., 2016). For example, using the direct methodology, Jia et al. (2016) utilized an advanced extrusion system and bio-ink blend to fabricate highly organized cell-loaded perfusable vascular structures. The coaxial nozzle system was capable of producing tubes with a wide range of diameters (500–1500 μm) and wall thicknesses (60–280 μm). In combination with bio ink containing encapsulated vascular cells, this method could induce formation of functional vessels (Jia et al., 2016).

Lately, focus has shifted toward the use of indirect bioprinting due to the higher level of precision and wider array of biomaterials that can be utilized. For example, Miller et al. (2012) printed a 3D filament network of carbohydrate glass and embedded it in a hydrogel. The filament network was then sacrificed to create a cylindrical network with controlled geometry which could then be seeded with ECs (Miller et al., 2012). Kolesky et al. (2014) used a similar sacrificial approach using a fugitive bio-ink composed of Pluronic F127 mixed with cell laden Gelatin-Methacryloyl (GelMA) to create a vessel matrix. This was then encased in a GelMA hydrogel and the bio-ink removed by liquifying, leaving a lumen which was then functionalized by seeding with ECs (Kolesky et al., 2014). More recently they have built upon this technique to produce vascularized tissues >1 cm in thickness with long term survival (Kolesky et al., 2016). This demonstrates the potential of 3D bioprinting for creating physiologically relevant vascularized tissues.

Laser-based bioprinting is a less common form of bioprinting and is performed via either photopolymerisation or laser-induced forward transfer (Sasmal et al., 2018). Although expensive, this technique can print cells at a very high resolution without subjecting them to shear stress (Kant and Coulombe, 2018). Wu and Ringeisen (2010) demonstrated the feasibility of this approach by using biological laser printing (BioLP) to fabricate human umbilical vein endothelial cell (HUVEC) and smooth muscle cell (SMC) branch structures with interconnected lumens. Furthermore, Jia et al. (2016) recently utilized a photopolymerisation-based 3D bioprinting system to create a complex pre-vascularized tissue. Assessment of the tissue 2-weeks post implantation revealed successful anastomosis between tissue and host vasculature. In addition, a significant increase in vascular density and number of vessels was observed in the pre-vascularized tissue, as compared to the control.

Bioprinting is an exciting field that holds great potential for delivering controlled angiogenesis. Studies have already demonstrated success in promoting vascularization in engineered tissues with increasing scales. With advancements in printing technologies to increase precision of printing, and the development of less expensive techniques, there is no doubt that we will see printable pre-vascularized whole tissues in the years to come.

#### Electrospinning

In recent years, researchers have explored the possibility of using electrospinning techniques to produce nanofiber-based vascular networks. This fabrication technique allows for fine control over properties such as diameter, porosity and degradation rate. In addition, fibers produced using this method possess a similar diameter to natural ECM (50–500 nm) and therefore mimic natural topographical cues (Jundziłł et al., 2017). Kenar et al. (2019) created a micro-fibrous composite scaffold using poly(L-lactide-co-ε-caprolactone) (PCL) blended with collagen and hyaluronic acid that greatly enhanced the length of vasculature in engineered tissues. Furthermore, Cui et al. (2017) demonstrated improved pre-vascularization of constructs by seeding HUVEC on LBL aligned (PCL)/cellulose nanofiber matrices. Once implanted, the aligned fiber matrices promoted integration with the host vasculature. Other groups have used the nanoscale properties of electrospinning to produce matrices capable of mimicking bone ECM and significantly enhancing *in vivo* angiogenesis via spatial organization of fibers (Gao et al., 2017), further highlighting the potential as a vascularization strategy.

### Patterning of Bioactive Molecules

The biofabrication methods described above can be also employed to pattern bioactive molecules within scaffolds, in an attempt to emulate biochemical gradients, present in natural angiogenesis, and promote *in situ* vascularization via integration with the host vascular network.

Due to its potent effect on angiogenesis, VEGF is the most commonly used growth factor for patterning of scaffolds. For example, Alsop et al. (2014) used photolithography to print VEGF onto a collagen-glycosaminoglycan scaffold in a spatially defined manner. They reported greater cell infiltration into the scaffold and presence of immature vascular networks. Similarly, hydrogels have been designed that induce directional vessel growth via pre-defined release of VEGF (Rich et al., 2014). The requirement for precise spatial control is demonstrated by the observation that promotion of aligned vasculature was only detected when the hydrogel was printed parallel to existing vasculature, but not when oriented perpendicularly.

In addition to spatially defined GF deposition, some researchers have incorporated combinations of different GF into the scaffold material to better replicate the different stages of angiogenesis (Rouwkema and Khademhosseini, 2016). Improved angiogenesis and maturation of the construct has been reported both with a combination of VEGF, FGF, and BMP2 (Kuttappan et al., 2018), and with VEGF and Angiopoietin (Chiu and Radisic, 2010).

Even with the use of multiple GF, these systems are still relatively basic in comparison to the complexity of native angiogenesis. The simple incorporation of bioactive molecules in the scaffold material does not sufficiently ensure its spatial localization, due to diffusion and burst release (Rouwkema and Khademhosseini, 2016; Kant and Coulombe, 2018). In order to address these challenges, various techniques have been employed. Wu et al. (2016) functionalized decellularised scaffolds with heparin via end point attachment. This enabled the scaffolds to bind and release heparin-binding GF, such as VEGF, with an increased level of control, resulting in enhanced angiogenesis. Another group printed a biodegradable polymer scaffold with conflicting zones of VEGF and VEGF inhibitors to spatially restrict signaling (Yuen et al., 2010).

In addition to GF, peptides have been used to functionalize scaffolds and induce vessel formation. These peptides can incorporate angiogenic domains whilst possessing a greater stability than GF (Fu and Wang, 2018). For example, Shu et al. (2015) demonstrated that functionalization of hydrogels with RoY, a 12 amino acid synthetic peptide, increased tube formation *in vivo* compared to the non-functionalized scaffold. Similarly, incorporation of peptide motives KLT and PRG into peptide nanofiber scaffolds resulted in a positive effect on endothelial cell viability and proliferation (Wang et al., 2011). Interestingly, Lei et al. (2012) used photolithography to micropattern SVVYGLR peptide strips on polymer surfaces. ECs seeded onto 10 and 50 μm peptide strips were directionally regulated and underwent morphogenesis, forming tubular structures with a central lumen. Conversely, tube formation was not observed in larger, 100 μm strips. This underlines the importance of spatial organization in vascularization strategies.

Many other peptide sequences have also been used to improve adhesion of ECs, which in turn encourages vessel development (West and Moon, 2008). Importantly, peptides are very easily patterned on to surfaces and scaffolds by covalent binding to the scaffold material, and are relatively resilient to processing. These characteristics have been harnessed for vascular TE by electrospinning two differently peptide-conjugated PCL solutions. The produced scaffold presented spatially organized functionalities, the SVVYGLR EC adhesion peptide and a heparin-binding peptide, and displayed improved endothelialisation (Campagnolo et al., 2016). Similarly, Chow et al. (2014) utilized peptide-PCL conjugates with a specific affinity for glycosaminoglycans (GAGs), combined with sequential electrospinning techniques, to guide spatial organization of GAGs throughout a scaffold. Using this method, rather than covalent bonding, preserves the bioactivity of the GAGs, mimicking the natural ECM and providing a more clinically relevant tissue construct. Both the heparin-binding peptide and the GAG gradient within the scaffolds help to organize growth factor and cytokine distribution, promoting cell infiltration and direction of biological processes.

Patterning of biomolecules shows great promise for promoting vascularization in engineered scaffolds and tissues. Researchers have begun to address initial limitations, such as random distribution of GF, by encapsulating biomolecules before patterning of scaffolds. There is still a long way to go before true chemotactic gradients representative of the *in vivo* angiogenic environment can be realized. Technologies such as nanoparticles, which provide a greater level of control for biomolecule patterning and release, offer great hope for the future.

## Interaction with the ECM

The microenvironment represents a fundamental regulatory system for angiogenesis, and the analysis of cell-ECM interactions is an important area for TE research.

The main challenge for TE is to develop a biologically inspired scaffold to mimic the natural ECM. For this purpose, natural polymers, such as hydrogels, represent the preferred choice because of their biomimetic potential (Fonseca et al., 2014). However, reproducing the complexity of the matrix is not easy and a series of factors must be considered; such as mechanical properties, cell adhesion and incorporation of GF.

## Mechanical Properties of the Scaffold

The mechanical properties of scaffolds, i.e., stiffness, porosity, shear stress, hydrophobicity, and internal architecture (alignment), have been shown to affect cellular behavior *in vitro*. ECs are capable of modulating their behavior in response to alterations in substrate stiffness. For example, HUVECs can increase their expression of MMPs, such as MMP2, MMP3, MMP4, and MMP14, and of angiogenic GF, such as VEGFA, when co-cultured *in vitro* with adenocarcinoma cells on stiff polydimethylsiloxane (PDMS) substrates, whilst MMPs are downregulated with decreased stiffness (Zhao et al., 2018). Increased stiffness of polyacrylamide (PA) scaffolds from 1 to 10 kPa has been shown to boost the endothelial cell response to VEGF by increasing VEGFR-2 internalisation (LaValley et al., 2017). Similarly, increased stiffness of collagen coated PA gels in a range from 3 to 3,000 Pa affected the expression of functional proteins and GF in HUVECs, but did not affect their proliferation and gene expression (Santos et al., 2015). Differentiation of endothelial progenitor cells was proportional to the stiffness of the PDMS scaffolds (Xue et al., 2017). Despite advancements in unveiling the cell-sensing of stiffness, the molecular signaling remains unclear. Furthermore, during migration and sprouting, cells can experience different stiffnesses, which is also dependent on the tissue type (Singh et al., 2018).

The porosity level, pore size and pore interconnectivity of a scaffold all affect cell behavior considerably (Loh and Choong, 2013; Bružauskaitë et al., 2016). For example, a pore size increase (to >40 μm) in PCL scaffolds was shown to enhance colonization and tube formation of endothelial progenitor cells (Hong et al., 2015). Likewise, cardiomyocytes demonstrated increased survival, enhanced angiogenesis and reduced fibrotic reaction in poly(2-hydroxyethyl methacrylate-co-methacrylic acid) hydrogel scaffolds of 80 and 40 μm pore size, as compared to 20 μm and non-porous constructs (Madden et al., 2010). In general, a minimum overall porosity of approximately 50%, along with a pore size of approximately 35–100 μm, is considered optimal for blood vessel formation (Oliviero et al., 2012). Mathematical simulations enable a more accurate design and prediction of the optimal pore size for maximizing homogenous cell distribution and cell proliferation within a specific scaffold (Mehdizadeh et al., 2013).

Furthermore, the scaffolds’ surface topography and physical/chemical properties can affect cell behavior. For example, changes in wettability and electric charges can affect cell adhesion on a biomaterial surface (Guo et al., 2016). From a topographical point of view, the roughness, i.e., nano-roughness (<100 nm), micro-roughness (100 nm–100 μm), and macro-roughness (100 μm–1 mm), and curvature of a surface can affect cell proliferation, spreading and metabolic activity (Ferrari et al., 2019). Roughness of scaffolds at the nano-range have shown to improve the growth of ECs on poly-urethane-poly(ethylene glycol) surfaces (Chung et al., 2003).

Overall, whilst the above mechanical properties have been proven to affect cell behavior and angiogenesis *in vitro*, the exact biological mechanisms of action remain unexplored. Furthermore, different studies are conducted on biomaterials of different properties and are limited to a single parameter variation, ignoring the great challenge represented by the interdependency of these parameters. For example, altering the porosity of a material alters its stiffness, and for this reason disentangling the individual effects from altering single mechanical parameters/factors is hugely complicated (Chim and Mikos, 2018). Overall, systematic studies altering multiple mechanical properties on several materials are still needed to robustly unravel the impact on angiogenic cell behavior.

### Promotion of Cell Adhesion

Attachment of cells to a scaffold, followed by migration in the pores and proliferation, is necessary for the correct development of a 3D structure. The cell-ECM interaction is mediated by transmembrane proteins, such as integrins, exposed on the cell surface (Post et al., 2019). Their key role is to initiate an intracellular signaling cascade that induces the cell to attach, migrate or differentiate (Ivaska and Heino, 2000). Many strategies have been used to facilitate this process by coating the scaffolds with proteins like collagen, laminin, and fibronectin (Post et al., 2019). Stamati et al. (2014) demonstrated that by coating a collagen I hydrogel with laminin, EC aggregation patterns can be regulated (Stamati et al., 2014). In the presence of laminin, EC formed an end to end network with increased integrin α6 expression and VEGF uptake. However, in absence of laminin EC formed cobblestone-like sheets and slowed VEGF uptake (Stamati et al., 2014). Importantly, by changing the chemistry, density and composition of peptides on the backbone, it is possible to control the stiffness of the biomaterial independently from the scaffold architecture (Spicer, 2020).

Covalent immobilization of peptides/proteins on the surface of scaffolds can be used as an alternative to coating, improving the stability of biomolecules (Zhu and Clark, 2014) and allowing the selection of cell-specific sequences to target the desirable cells on the scaffold (Uchida et al., 2007). John et al. (2019) fabricated a GelMA nanofiber microsphere coated with VEGF-mimic peptides, and showed the formation of vascular tubes by HUVEC. Similarly, Flora et al. (2019) demonstrated that elastin-like recombinamer (ELR) tethered with VEGF-mimic peptides not only improved vascularization *in vitro*, but also *in vivo*. In fact, ELR injected into mice enhanced the recruitment and proliferation of ECs, and the number of capillaries (Flora et al., 2019).

### Presentation of Growth Factors

Soluble GF often contain sequences targeting the ECM, which *in vivo* allows the correct presentation of the GF and provides the spatio-temporal definition to the gradient. When designing a 3D matrix, the simplest way to incorporate GFs is to encapsulate them during gelation or solidification (Wang et al., 2017b). However, in order to improve the exposure of the GF on the fibrils, effective results are obtained using self-assembling peptides (King and Krebsbach, 2012). Rajangam et al. (2006) used heparin-binding peptide amphiphile nanostructures containing heparin binding sites for GF like VEGF and FGF-2. Interestingly, the rigidity of the scaffold allows the GF to expose the bioactive domains, minimizing Brownian motions and promoting cell attachment and receptor binding (Rajangam et al., 2006). Similarly, Chow et al. (2010) used nanoscale fibers to deliver VEGF and FGF-2 to pancreatic islets, enhancing the bioactivity of the GF, promoting the angiogenic sprouting and increasing islet survival, providing new insights into *in vivo* therapeutic applications. In addition to engineering the scaffold, the affinity of vascular GF to the ECM can be improved by means of protein engineering. An example of this is the fusion of the strong ECM-binding domain of the placenta growth factor-2 protein, which was shown to enhance the repair of chronic wounds and bone defects (Martino et al., 2014).

## Other Mechanisms

### Effect of Circadian Clocks

Circadian rhythms modulate many physiological processes and are essential for the maintenance of homeostasis within a tissue. The clock machinery has been identified in vascular cells (Nonaka et al., 2001; Takeda et al., 2007), bringing attention to the effect of the circadian clock in vascular formation and remodeling. Particularly, the peripheral circadian clock in the vasculature influences the formation of new blood vessels *in vivo* (Jensen et al., 2014). A disrupted circadian clock leads to pathological, uncontrolled angiogenesis (Anea et al., 2009; Jensen et al., 2014; Block, 2018). Moreover, the effect of circadian clocks on the cell specific response to micro-environmental changes has been examined. Interestingly, in the context of TE, circadian rhythms appear to be mechano-sensitive (Streuli and Meng, 2019). The structure and stiffness of the tissues, as well as the composition of the ECM, affects circadian output and appears to be cell-specific (Yang et al., 2017; Broadberry et al., 2018; Williams et al., 2018). This area still requires extensive research but, in the future, may develop new evidence toward circadian control of angiogenesis in cancer and wound healing. For a complete overview of the subject, we recommend the recently published article by Streuli and Meng (2019).

### Flow

The endothelial layer of blood vessels is constantly exposed to the blood stream and hemodynamic forces, such as shear stress and tangential forces (Song and Munn, 2011). Cells respond to these forces by changing their morphology and gene expression (Wragg et al., 2014), with shear stress inhibiting EC proliferation and limiting sprouting to low flow regions (e.g., tumor microenvironment and ischemia) (Song and Munn, 2011; Tun et al., 2019). Song and Munn (2011) developed a perfused microfluidic device and reported a reduction in VEGF-induced sprouting under physiological shear stress. Despite several microfluidic solutions developed to study angiogenesis (Yeon et al., 2012; Zheng et al., 2012; Motherwell et al., 2019), the current focus is limited to the evaluation of the platforms with limited application to molecular or biological studies.

The application of flow to enhance the maturation of blood vessels within TE scaffolds is still not fully developed. For example, dynamic flow and enhanced shear stress applied to endothelial cell seeded poly-(L-lactic acid) scaffold enhanced EC migration (Koo et al., 2014). Indeed, the flow derived Wall Shear Stress (WSS) affects the interaction between ECs (EC-EC), as well as between EC and smooth muscle cells (EC-SMC), in TE constructs, consequently affecting vessel maturation (Shav et al., 2014).

The next challenge for TE is to integrate this newly acquired knowledge to promote the formation of functional blood vessels in engineered scaffolds.

### Hypoxia

Low oxygen concentration is a potent regulator of angiogenesis, driving sprouting of ECs toward the deficient tissue (Pugh and Ratcliffe, 2003; Ziyad and Iruela-Arispe, 2011; Viallard and Larrivée, 2017; Petrova et al., 2018) through hypoxia inducible factor-dependent increase of VEGF transcription (Liao and Johnson, 2007; Oladipupo et al., 2011). Recreating a hypoxic environment has been revealed to be useful for therapeutic angiogenesis in bone TE both *in vitro* and *in vivo* (Wu et al., 2012; Deng et al., 2019). Wu et al. (2012) developed a porous cobalt-containing mesopore-bioglass scaffold able to release ionic Co^2+^, inducing a hypoxic-mediated response in human bone marrow stromal cells. Similarly, Deng et al. (2019) used a cobalt-doped bioactive borosilicate glass scaffold *in vivo*, showing improved vascularization and regeneration of bone tissue after implantation in rats with a calvarial defect.

## Interactions with Other Cells

Endothelial cells are the primary cell type driving angiogenesis, however, their use alone is often not sufficient to recapitulate physiological angiogenesis in TE applications (Costa-Almeida et al., 2014). Angiogenesis *in vivo* involves complex signaling pathways between ECs and other associated supportive cells, such as pericytes and macrophages (Ribatti and Crivellato, 2009; Cathery et al., 2018). In order to mimic this natural process, researchers have used combinations of proangiogenic cells to promote the vascularization of scaffolds. Here, we discuss the cell types which have shown potential for this purpose.

### Heterogeneity of Endothelial Cells

All vascular EC originate in the embryonic mesoderm (Dyer and Patterson, 2010), however, they display remarkable organ-specific characteristics and genetic programming, leading to specialization in morphology, such as fenestrations, and functionality, such as cell-cell interaction and permeability (Chi et al., 2003; Nolan et al., 2013). EC differentiation relies on both epigenetic mechanisms and the interaction with the microenvironment, determining the existence of different sub-types of EC even within the same vascular bed (Kusumbe et al., 2014; Ramasamy, 2017; Barry et al., 2019). For example, two sub-populations of EC have been characterized in the murine skeletal system; the H type, which are responsible for angiogenesis, and the L type, which form the sinusoidal capillaries in bones (Kusumbe et al., 2014). Interestingly, when EC are extracted from their natural environment and cultured *in vitro*, they can lose their specialized gene expression signature (Lacorre et al., 2004). One meaningful example is the reduced ability of brain ECs to independently recapitulate the impenetrability of the blood-brain barrier in culture (Janzer and Raff, 1987; Dyer and Patterson, 2010). This heterogeneity must be considered when selecting the EC population to be employed for organ-specific TE applications.

### Perivascular/Mural Cells

Perivascular cells, such as pericytes, vascular smooth muscle cells (VSMCs), mesenchymal stromal cells (MSCs) and fibroblasts reside within the vascular niche and play an integral role in blood vessel development (Costa-Almeida et al., 2014). Pericytes contribute to angiogenesis and vessel maturation through direct contact with ECs and secretion of GF, MMPs, and ECM deposition (Cathery et al., 2018). Fibroblasts secrete a similar array of potent angiogenic factors and MMPs, and are also the primary source of ECM, whilst VSMC play a similar supportive role in larger blood vessels (Costa-Almeida et al., 2014). For this reason perivascular cells have become the preferred choice to support vascularization in TE (Avolio et al., 2017). For example, Wang et al. (2017a) demonstrated that the addition of pericytes to a co-culture system seeded into a calcium phosphate cement scaffold increased vessel density by over 65% after 12 weeks. Sathy et al. (2015) also reported promising results following incorporation of pericytes into multi-layered scaffolds designed for tissue engineered bone. The authors were able to produce vascularized bone constructs of up to 400 μm in thickness. Recently Liu et al. (2019) utilized a co-culture system of ECs and bone marrow MSCs encapsulated in Gel-MA microspheres to investigate whether MSCs could mimic the role of pericytes and increase angiogenesis. They discovered that the MSCs differentiated toward a pericyte phenotype, and subsequently significantly enhanced vascularization and integration with host tissue *in vivo* (Liu et al., 2019). Although pericyte-like cells seem like the popular choice due to their prominent role in angiogenesis, some research groups have also reported success with other perivascular cells. Wang et al. (2012) observed rapid vascularization of tissue engineered grafts *in vivo* using a scaffold seeded with ECs and VSMCs. Likewise, Guerreiro et al. (2014) demonstrated that fibroblasts immobilized in alginate microspheres support assembly of capillary-like structures. Overall, these findings demonstrate the suitability of perivascular cells for angiogenesis in TE. Their easy isolation combined with their close association with blood vessels make them an ideal candidate for further studies.

### Macrophages

Immune cells, such as macrophages, are closely connected with angiogenesis. Macrophage phenotypes vary along a spectrum from a pro-inflammatory M1 state to a pro-healing M2 state (Moore and West, 2019), resulting in certain subsets of macrophages triggering inflammation, and others tissue healing, after injury. Pro-healing macrophages act by secreting pro-angiogenic factors that promote endothelial proliferation and migration, resulting in neovascularization (Ribatti and Crivellato, 2009). Moreover, macrophages play a key role in vascular anastomosis, which further highlights their potential for use in the vascularization of tissue engineered constructs (Fantin et al., 2010). In fact, Spiller et al. (2014) proposed that a combination of activated macrophage phenotypes could be used to control angiogenesis in TE strategies. By modifying scaffold properties, they were able to direct macrophage phenotype transitions and increase vascularization. Similarly, Barthes et al. (2018) incorporated phenotype-restricted macrophages into a 3D gelatin hydrogel scaffold. They reported that the presence of macrophages not only amplified the angiogenic microenvironment, but also gave rise to highly organized sprouting that resembled capillary-like structures. Additionally, Moore et al. (2018) used a bioactive PEG-based hydrogel to study the role of various macrophage phenotypes in vessel development. The authors discovered that whereas some phenotypes enhanced tube formation in tissue engineered scaffolds, others had an antagonistic effect. This demonstrates that whilst macrophages have shown potential for tissue vascularization strategies, caution must be applied to avoid adverse effects.

### Neural Cells

Similarities between the nervous and vascular systems have been well studied. These systems align with each other throughout the body and develop highly branched networks through common cellular and molecular principles (Eichmann and Thomas, 2013). In fact, the specialized ECs termed “tip cells,” which lead the vascular sprouting process, express receptors for axon guidance molecules. Loss of function of these receptors results in defective vessel formation, showing their importance for angiogenesis (Eichmann and Thomas, 2013). Himmels et al. (2017) recently described a neuro-vascular signaling mechanism responsible for guiding vessel development. The authors demonstrated that motor neurons regulate blood vessel patterning via an autocrine mechanism involving VEGF secretion and expression of a VEGF trapping receptor, soluble fms-like tyrosine kinase-1 (sFlt1) (Himmels et al., 2017). Furthermore, Emanueli et al. (2003) described the intricate role neurotrophins play in vascularization. In a mouse model of limb ischemia, overexpression of neurotrophin-3 (NT-3) was shown to increase capillary and arteriolar density (Cristofaro et al., 2010). Similarly, *in vitro* studies showed NT-3 stimulated endothelial network formation, increasing the number and diameter of functional vessels. This research further highlights the potential of neural derived cells and GF for vascularization strategies.

Within a TE context, Rauch et al. (2009) showed that the addition of neural progenitor cells to composite polymer scaffold seeded with ECs, resulted in a two-fold increase in functional vessels. Ford et al. (2006) demonstrated similar results with neural progenitor-endothelial cocultures seeded in microporous scaffolds, producing a functional microcirculation *in vivo*. Although there are limited studies to date, these discoveries suggest that integration of neural-derived cells into engineered tissue constructs could help guide angiogenesis and vascular development.

## Conclusion and Perspectives

**FIGURE 1:**
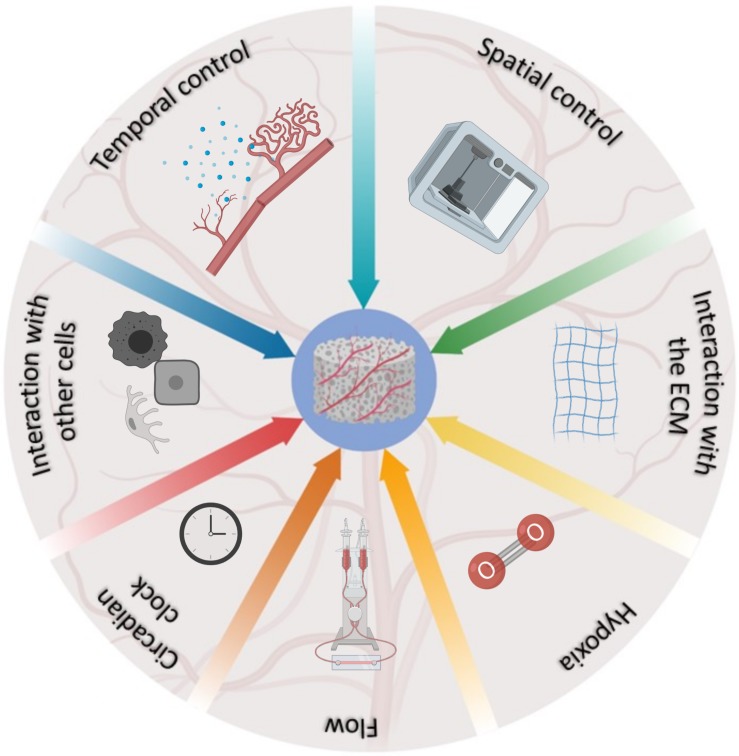
Schematic representation of the factors involved in the development and regulation of blood vessels in vivo, and their relative contribution as represented by the fraction occupied in the pie chart.

This review provides a summary of the most advanced bioengineering solutions developed to mimic the natural development of blood vessels within TE constructs, and the complex network of interacting factors regulating this phenomenon *in vivo* (Figure 1). It is indisputable that scientists are increasingly more aware of the complexities of the phenomenon and that the landscape has progressively become more articulated. However, we are still in the initial phases of this process and the development of more complex scaffolds, encompassing several of the characteristics described above, is a desirable avenue for future studies. Undoubtedly, the development of deep-learning algorithms through machine learning (Park et al., 2018; Nguyen et al., 2019) can potentially open a new era for the multiparametric acquisition of data, offering a window on the resolution of TE vascularization, as nature intended.

## Author Contributions

VM: writing, reviewing, and figure making. WC and EV: writing and reviewing. PM: planning and reviewing. PC: planning, writing, reviewing, and coordinating.

## Conflict of Interest

The authors declare that the research was conducted in the absence of any commercial or financial relationships that could be construed as a potential conflict of interest.
